# Staff Experiences at a New York City Medical Center During the Spring Peak of the Covid-19 Pandemic: A Qualitative Study

**DOI:** 10.21203/rs.3.rs-268807/v1

**Published:** 2021-03-24

**Authors:** Liz Blackler, Amy E. Scharf, James N. Masciale, Kathleen A. Lynch, Jamie C. Riches, Konstantina Matsoukas, Michelle Colletti, Lisa Wall, Sanjay Chawla, Nessa Coyle, Yesne Alici, Rebecca Guest, Louis P. Voigt

**Affiliations:** Memorial Sloan Kettering Cancer Center; Memorial Sloan Kettering Cancer Center; Memorial Sloan Kettering Cancer Center; Memorial Sloan Kettering Cancer Center; Memorial Sloan Kettering Cancer Center; Memorial Sloan Kettering Cancer Center; Memorial Sloan Kettering Cancer Center; Memorial Sloan Kettering Cancer Center; Memorial Sloan Kettering Cancer Center; Memorial Sloan Kettering Cancer Center; Memorial Sloan Kettering Cancer Center; Memorial Sloan Kettering Cancer Center; Memorial Sloan Kettering Cancer Center

**Keywords:** Health professionals, Hospitals, COVID-19, Psychosocial, Coping, Qualitative

## Abstract

**Purpose:**

In March-April 2020, New York City was overwhelmed by COVID-19 infections, leading to substantial disruptions in nearly all aspects of care and operations at most local hospitals. This qualitative study of a quaternary, urban oncology hospital investigated the effects of these disruptions upon a professionally diverse cohort of its employees, including physicians, nurses, respiratory therapists, pharmacists, security guards, histology technicians, and environmental services workers.

**Methods:**

The participant pool were selected through a combination of purposive and random sampling methodology and coders performed a thematic content analysis of open-ended responses.

**Results:**

Analysis revealed several important themes, including concerns about exposure for self and others; patient care as a source of both satisfaction and stress; psychological consequences of uncertainty and ambiguity; family as sources of both comfort and apprehension; the importance of adequate institutional communication; and support from colleagues.

**Conclusion:**

Results and analysis provide suggestions for institutional policies and initiatives in the event of a COVID-19 surge or another public health crisis. Administrative efforts should aspire to establish, strengthen, and promote interdisciplinary and interdepartmental efforts to address, and mitigate workplace and personal stressors. through timely and transparent communications, consistent clinical guidance and information about changes in hospital policies and supplemental employee assistance.

## Introduction

The COVID-19 pandemic has presented multiple societal and disease-related challenges not seen since the historic influenza outbreak of 1918. Globally, nearly all areas of daily life were disrupted by COVID-19, e.g., physical and mental health, healthcare delivery, government, business, law, education, the economy, and social interactions. The speed and magnitude of COVID-19’s impact and the subsequent governmental and institutional responses were, undoubtedly, stressors for much of humanity around the world [1–3].

During the early weeks of the COVID-19 pandemic in New York City (NYC), our quaternary cancer center, Memorial Sloan Kettering (MSK), faced significant challenges as it strove to continue providing highly-specialized, time-sensitive, and effective oncology care while simultaneously treating SARS-CoV-2-positive cancer patients and protecting non-infected patients and staff from the virus. We hypothesized that employees at all levels – senior management, clinicians, administrators, and support staff – were substantially impacted, both positively and negatively, by their experiences working in a healthcare setting in the midst of the COVID-19 pandemic [4].

The purpose of this study was to assess how employees who provided direct and remote care to cancer patients diagnosed with COVID-19 reacted to an unprecedented pandemic situation. We sought to gain a deeper understanding of how MSK staff experienced the challenges of fulfilling their responsibilities to provide optimal care for a large number of critically ill and vulnerable patients, while also confronting concerns about resource scarcity, virus exposure, and inadvertently infecting family, friends, and others. Through a qualitative survey featuring a series of open-ended questions, staff from a variety of disciplines, responsibilities, and departments were asked to share their experiences on the frontlines. The information gained may help MSK and other healthcare organizations identify well-received initiatives and unmet needs which can then be addressed with immediate and long-term interventions. Insight also may be gleaned to better prepare for future public health emergencies affecting healthcare delivery systems.

## Methods

### Recruitment and Participants

The participant pool comprised staff who delivered direct care to patients diagnosed with COVID-19 or who were involved in coordinating MSK’s response to the COVID-19 crisis. Participants were selected via a combination of purposive and random sampling methodology. Eligible participants were recruited from various categories: Advanced Practice Providers (APPs), Environmental Services (i.e., housekeeping), Food and Nutrition, Attending Physicians, Histology Technicians (i.e., morgue employees), Nurses, Respiratory Therapists, and Security Officers. From an overall participant pool of 2698, 300 employees were randomly selected using the Surveyselect procedure in SAS statistical software (SAS Institute, 2017) and they were sent an invitation to complete the survey [5].

The initial recruitment emails were sent on June 5, 2020. Reminder emails were sent June 13 and June 22, with the survey period ending on June 26, 2020. We also requested the assistance of department directors in raising their teams’ awareness of the survey. All employees were afforded access to MSK on-site technology and time during their shifts to complete the survey, if they so wished. Employees were also afforded the opportunity for a phone or videoconference interview, during which one of the investigators would record and transcribe their answers. Each of these participants would have access to his/her transcripts to ensure accuracy, after which the responses would be uploaded anonymously into the Research Electronic Data Capture (REDCap) and the voice recordings and the transcribed texts would be destroyed [6]. No participants requested this support.

Collected data were anonymous, no identifiable information was collected, and participants’ answers could not be traced back to them. All responses were stored in REDCap.

### Setting

MSK is a 514-bed academic National Cancer Institute (NCI) designated specialty cancer hospital in NYC. During the COVID-19 pandemic, MSK cared for both established patients with cancer and patients with cancer who were referred from neighboring hospitals – regardless of their COVID-19 status. The majority of patients transferred from the nearby hospitals tested negative for COVID-19. Between March 10, and May 7, 2020, 946 patients tested positive for COVID-19 and 40% were hospitalized [7].

### The Survey

The 19-item survey consisted of a mix of demographic and open-ended questions. Seven open-ended questions were designed to encourage participants to reflect on their experiences as employees during the COVID-19 pandemic. Preceding the queries, participants were shown a prompt consisting of an image and short paragraph (see Appendix A1 for prompt and the full list of narrative questions). The image was taken from a *New Yorker* magazine cover from April 6, 2020 [8]. The intended purpose of this prompt was to provide a catalyst for participants to reflect on the COVID-19 pandemic and its effect on their professional and personal lives.

### Analytic Strategy

We calculated descriptive statistics for the demographic data, using SAS 9.4. (SAS Institute, 2017) [5]. We performed a thematic content analysis of open-ended responses using NVivo 12.0 [9, 10]. First, four members of the study team (KL, JM, AS, LB) independently coded participant responses to each question, using a set of *a priori* descriptive and interpretive codes and inductively generated codes that emerged from the common patterns in the data. After a question was independently coded, the study’s qualitative methods specialist (QMS) merged individual coding files and held a team meeting, where discrepancies in coding were discussed and statements were assigned to categories through consensus [11]. This process was repeated for each question, after which the QMS conducted a final quality assurance check to ensure fidelity to the codebook. Codes were then grouped into categories to facilitate identification of major themes per question and recurrent themes across the dataset.

Using the “Crosstab” function in NVivo [9], the team conducted a secondary analysis to assess whether code frequencies per question differed by selected demographic variables (e.g., role, tenure at MSK, gender). These data were exported to SAS and tested for statistical significance using the Chi-Square test with all *p* values of < 0.05. An ANOVA was performed to test whether there were systematic differences between the participants who answered the open-ended questions compared with those who did not, with the number of questions answered as the dependent variable and the demographics as independent variables. Tukey HSD was used as the alpha correction for between group mean comparisons.

## Results

Our analyses focused on open-ended questions 2 through 6 (see Online Resource 1). Themes emerging from Questions 1 and 7 were much more general and respondents interpreted these as unrelated to the current pandemic. (See Online Resource 2 for example responses).

Preliminary Chi-Square and ANOVA analyses determined that missing responses/skipped questions did not occur in any systematic pattern based on any of the demographic variables we queried (all *p*’s > 0.05). In other words, any skipped questions were the result of random variation among participants.

### Demographics

Our final sample consisted of 72 participants, yielding a response rate of 24%. Among the participants, 66% (n=48) were women, 81 *%* (n=58) worked predominantly daytime shifts, and 83% (n=60) worked on weekdays. Of note, 91% (n=65) did not test positive for COVID-19, but 83% (n=60) knew a colleague who did. A variety of professional roles was represented, with physicians and nurses each accounting for 14% of our sample (n=10 for each group; see Table 1). Table 2 illustrates the length of MSK tenure among participants, with one third having been at MSK for 3 years or less. Two-thirds of the respondants (n=48) also indicated that they interacted in person with cancer patients diagnosed with COVID-19 (see Table 3).

### Overall Themes

Across the open-ended questions, staff narratives of their experiences during COVID-19 coalesced into four major themes:

### Theme 1: Concerns about exposure and infection for self and others

Exposure to the virus emerged as the primary concern and stressor for staff working during the COVID-19 pandemic. Across all questions, participants reflected on the tension between balancing their responsibilities to patients with personal fears of infection – compounded by concerns over the availability of effective personal protective equipment (PPE). These fears were magnified by the novelty of the virus: information about COVID-19 changed in real time, making it difficult for participants to assess their own safety and exposure risks, and heightening their concerns over becoming virus vectors and infecting their loved ones. When asked to describe the “most difficult” part of their work during the pandemic (Question 3), 80% (40/50) of responses were related to concern about their own, their colleagues’ and their families’ exposure to COVID-19:

- “I was constantly worried though that I would get COVID, and that even if I didn’t show symptoms that I would have it and pass it on to my elderly parents”
- “I had nightmares of being infected or potentially infecting others, I prone myself every night as a result of fear of contracting this virus.”

### Theme 2: Patient care as a source of both meaning and stress

A positive sense of responsibility towards patients framed participants’ answers about their experiences. When asked to describe the “most gratifying” part of their work during the pandemic (Question 4), 87% (41/47) wrote about seeing or helping patients. Participants wrote that providing patient care and support helped them feel that they were making a difference and gave them a sense of direction, meaning and fulfillment during a time that felt out-of-control. Multiple participants also described the gratifying experiences of seeing patients recover from COVID-19. Additionally, in Question 4, 60% (28/47) of participants expressed gratitude for their employment, including statements of pride and professional satisfaction in being able to contribute to the pandemic response. Other responses conveyed appreciation for having a job during such an economically tumultuous period.

- “The most gratifying part of work during the pandemic is making sure the patients have a clean room.”
- “Seeing patients recover in the ICU and facetiming their families so they could see their loved ones off the ventilator.”

However, caring for patients was also described as a source of stress, frustration and sorrow. For example, when asked to describe the “most difficult” part of their work during the pandemic (Question 3), 48% (24/50) of respondents focused on the challenges of patient care. Many expressed helplessness in trying to manage patients infected with SARS-CoV-2, guilt in enforcing restricted visitor policies combined with difficulties in communicating with families, and frustration in trying to provide high-quality care remotely.

- “The most difficult part of work during this difficult time was the lost [sic] of patients.
- “It’s been difficult to be unable to perform an in-person assessment...you call a patient and they sound unwell, yet they do not want to go into UCC or seek escalated care.”
- “The most difficult part of my work during the pandemic was explaining to family members they could not see their gravely ill mother, father, child, etc.”

### Theme 3: Coping with Unknowns, Uncertainties, and Disruptions to their Lives.

Respondents experienced constant stress and anxiety as they attempted to navigate COVID-19-related professional and personal uncertainties. When asked to discuss the “weight of the COVID-19 pandemic on you, personally” (Question 2), 59% (30/51) of participants related concerns about their families - including virus exposure, their children’s physical and emotional health, and the ability to provide financial support. These stressors were compounded by the disruption of familiar routines and coping mechanisms. Across questions, many participants expressed the tension between their increased reliance on family for social and emotional support (especially as physical/social distancing increased) and the need to protect their loved ones from virus exposure. For some, work life and home life began to blend, making it increasingly difficult to juggle competing responsibilities. As a corollary, participants whose direct exposure to COVID-19 patients compelled them to quarantine away from loved ones described the difficulty and sacrifices they experienced.

- “I would distance myself from family member’s as everyday I feared bringing home Covid [sic].”
- “The most difficult part was dealing with the unknown, which generated a high level of fear. It took a lot of energy to stay calm and mentally sharp.”
- “There were changes in practice happening every day or sometimes by the hour...”

### Theme 4: Importance of Institutional & Professional Support:

Despite challenges, participants emphasized that professional collaborations were vital to helping them get through this difficult period. When asked what they found “the most helpful,” (Question 5), 70% (33/47) described positive interactions with colleagues, staff, and managers who were invested in both protecting co-workers from unnecessary exposure and providing meaningful social support.

- “I am also grateful for [my supervisor’s] support. When I recovered from COVID, I was...given a remote assignment which allowed me to work from home. The stress of commuting into the city during the crisis was gone.”
- “Co-workers and MSK staff worked collectively and diligently in navigating through the difficult times while providing quality patient care.”

Responders further expressed gratitude towards MSK for helping them feel valued and supported. They enumerated the institution-wide actions and policies (such as transportation assistance) that MSK implemented at the beginning and throughout the pandemic to mitigate the crisis-related stressors they were facing. When asked *“What could MSK have done or do now to make this stressful period easier or more manageable for you?”* (Question 6), 37% (16/43) responded that they believed there was nothing the institution could have done better to handle the pandemic and mitigate its effects on staff:

- “I talk to friends and ex-colleagues and I know that our organization did everything it possible [sic] could for the comfort of its employees.”
- “MSK out did [sic] themselves by offering so may support to the staff in terms of transportation, housing, financial assistance, etc.”

Participants also described stressors regarding timely communication from leadership, particularly at the onset of the pandemic, when information about COVID-19 and related safety protocols were rapidly evolving. Some perceived that new information was not communicated simultaneously to all staff, leading to a sense that team members *“were not always on the same page.” A* few statements referred to respondents’ direct leadership/supervisor and institutional leadership. Several participants offered suggestions, which included clearer communication around staffing decisions and protocols, and adjustments to benefits and perks during the COVID-19 pandemic, such as instituting hazard pay (53%, 23/43):

- “The first couple of weeks MSK was following CDC recommendations which were clearly behind what data had shown in other countries... The communication and protocols improved with time.”
- “I felt at the beginning of the pandemic the hospital was slow to provide PPE to staff even those who had valid concerns.”
- “Hazard pay would have been a major benefit.... It would also show a greater sense of appreciation.”

## Discussion

Participants’ responses support our initial hypothesis that staff were profoundly impacted by their experiences working in an NCI-designated specialty cancer hospital during the May through June 2020 COVID-19 surge in NYC. The first cases were reported in NYC on March 1, 2020 and our hospital admitted its first COVID-19-positive patients within the next two weeks [12]. Like other healthcare workers, numerous MSK employees tested positive for COVID-19 and many witnessed families and friends become ill during the Spring 2020 surge. In March 2020, 772 out of 23,000 total MSK staff tested positive for SARS-CoV-2, increasing to 1,329 by June 2020. By late March 2020, NYC became an epicenter of the COVID-19 pandemic, representing 53% of cases in New York State and 15.7% of all cases nationwide. By the middle of May 2020, more than 50,000 patients with COVID-19 were admitted to a NYC hospital, representing 26% of the city’s total cases [13, 14]. National, regional and local media reported on surge capacity plans, and the New York State government pushed hospitals to expand their bed capacity [15].

Staffs’ responses reflected deep personal stressors, the most prevalent being fear about inadvertently infecting themselves and loved ones with COVID-19. These concerns dovetailed with anxieties over PPE accessibility. Such worries were not unfounded: early in the pandemic, many NYC healthcare organizations were challenged by PPE shortages [16, 17].^.^ MSK’s response to COVID-19 in general, and PPE specifically, was rapidly evolving during the Spring 2020 surge, and may have contributed these fears and expressions of frustration over communication inconsistencies. As knowledge and experience with COVID-19 progressed, MSK became better-prepared to provide sufficient PPE and expeditiously communicate COVID-related policies and procedures. Beyond fears of infection, respondents also recounted personal anxieties such as familial financial pressures, difficulties finding childcare, and social isolation. Despite these challenges, several respondents stated that their ability to work, to contribute in a meaningful way, and to provide patient care helped them cope with this stressful time. Similarly, many reported gratification in receiving some form of appreciation from patients, colleagues, managers, and MSK more generally.

On a professional level, our survey responses conveyed anxiety and concern over the ability to provide high-quality care for patients with cancer who were diagnosed with COVID-19. Many reported feeling overwhelmed by their inability to help patients overcome COVID-19, and these extended into clinicians’ concerns about adequately caring for patients remotely and/or via telemedicine. It is therefore necessary to devise and implement safe and effective institution-wide measures to ensure adequate COVID-19 management and the uninterrupted delivery of critical cancer care in the event of a surge or other public health crisis. These may involve contingency plans for reconfiguring inpatient and outpatient facilities in order to create physically-separated spaces for cancer patients with and without a concurring diagnosis of COVID-19 or other highly-contagious disease. In addition, the provision of patient care via telemedicine may be improved through enhanced platforms and comprehensive staff training and support for these technologies.

Institution-wide restrictive visitor policies were a source of stress for MSK staff, with many respondents expressing distress over having to keep cancer patients infected with SARS-CoV-2 and their families separated, specifically those who were receiving end-of-life care. Early in the pandemic, MSK and other institutions such as The Johns Hopkins Hospital introduced innovate means to bridge the physical chasm between patients and their loved ones by outfitting patient rooms with electronic devices which allowed for seamless video-conferencing [18]. These and other solutions were essential in providing greater emotional comfort and support to patients and families while also protecting the safety of clinicians and families.

### Limitations

Our institution’s COVID-19 surge experiences and the results of this survey may not be representative of or applicable to other institutions. MSK is singularly focused on the diagnosis, treatment and research of cancer, and consistent with its mission, during the pandemic, only admitted patients with COVID-19 who also had cancer diagnoses. Given this institutional focus, it may have been enlightening to directly ask participants how the pandemic impacted their ability to deliver cancer-directed treatment for patients with and without COVID-19 [19–21]. This question is most relevant to clinicians (physicians, nurses, etc.) who provide direct cancer therapies to patients, and therefore a separate survey of these healthcare professionals would be informative [22]. Our survey purposefully sampled a professionally heterogeneous cohort of MSK employees, many of whom may have found such an inquiry non-applicable or irrelevant.

Although our sample size enabled us to attain thematic saturation, a higher response rate (of 50% or more) may have provided a richer set of responses to help us understand the varied staff experiences during the COVID-19 pandemic. We cannot exclude the existence of some response bias, and since we have no demographic data for the entire cohort of staff who were invited to participate in our survey, we cannot determine whether systematic differences existed between those who responded and those who did not. Participants answered our survey questions in June 2020 – several weeks after NYC’s pandemic peak – potentially resulting in retrospective biases. However, had we offered the survey during the Spring 2020 peak, our response rate may have been even lower due to staff’s being overwhelmed with pandemic-related tasks and stressors.

A large-scale cross-sectional quantitative survey that investigates the themes identified in this study may ascertain and validate the sentiments expressed by our participants. Expanding such a study to other institutions and a wider array of employees may provide a more comprehensive understanding of the implications of the pandemic on the diversity of individuals working in the healthcare field.

## Conclusion

Through our qualitative study, we sought to gain a deeper understanding of how MSK staff who worked directly and indirectly with cancer patients diagnosed with COVID-19 reacted to the challenges they encountered during the early days of the pandemic. In preparation for further surges of COVID-19 or other public health emergencies, and given the themes this survey uncovers, we believe that administrative efforts should aspire to establish, strengthen, and promote interdisciplinary and interdepartmental efforts to address, and mitigate workplace and personal stressors as much as possible. At the forefront, institution-wide access to timely and transparent communications is necessary to alleviate uncertainty and bolster organizational solidarity. Staff will benefit from real-time, consistent clinical guidance and information about changes in hospital policies, procedures and resources. Supplemental employee assistance in the form of emotional, financial and/or caregiver supports may serve to lessen personal strains and pressures. We believe that the suggested measures may improve staff experiences in the event of a COVID-19 surge or another public health crisis.

## Supplementary Material

Supplement

Supplement
