# Supplementary material for: Staff Experiences at a New York City Medical Center During the Spring Peak of the Covid-19 Pandemic: A Qualitative Study
Source: Res Sq. 2021 Mar 24:rs.3.rs-268807. Preprint. [Version 1] doi: 10.21203/rs.3.rs-268807/v1 (PMC8010741; doi:10.21203/rs.3.rs-268807/v1)
Supplement: Supplement [file 13a7438912f693c703f6b20b.pdf]

Table 1: Characteristics of Survey Participants

**Characteristics**	**No. (%)**
Number of Participants	72
**Gender**	
Female	48 (66.7)
Male	24 (33.3)
**Distribution of Job Role**	
Clinical Roles	42 (58.3)
Nursing Assistant	1 (1.4)
Nurse Practitioner (NP)	8 (11.1)
Physician	10 (13.9)
Physician Assistant (PA)	6 (8.3)
Registered Nurse (RN)	10 (13.9)
Respiratory Therapist	7 (9.7)
Non-Clinical Roles	20 (27.8)
Administrator	1 (1.4)
Environmental Services	6 (8.3)
Food & Nutrition Services	5 (6.9)
Laboratory Assistant	2 (2.8)
Personal Care Technician (PCT)	1 (1.4)
Supervisor/Manager	4 (5.6)
Unit Assistant	1 (1.4)
Other	10 (13.9)

Table 2:

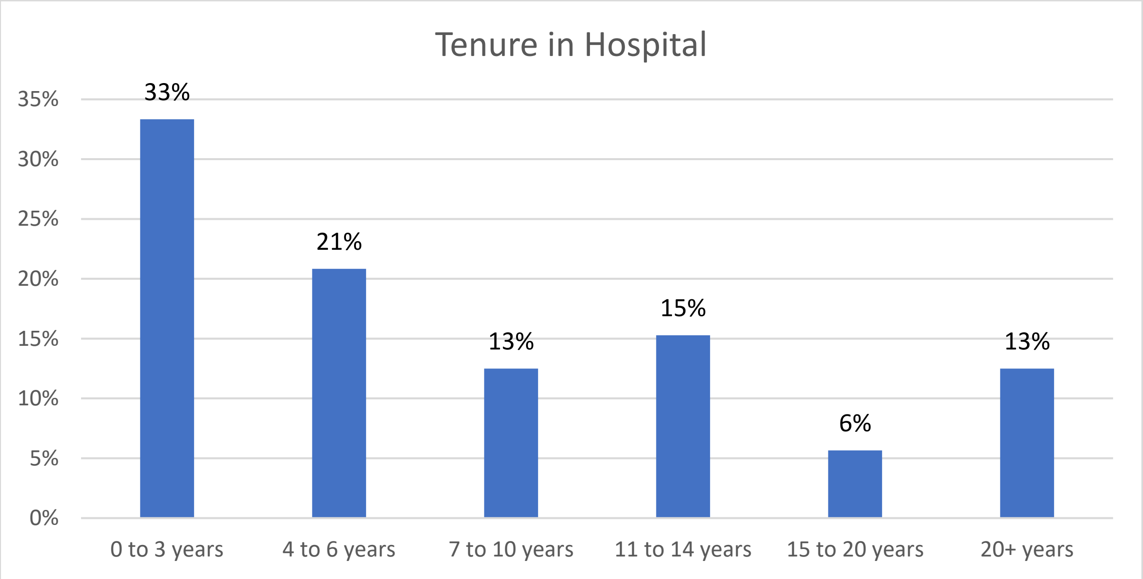

Table 2 Distribution of participants job tenure.

Table 3:

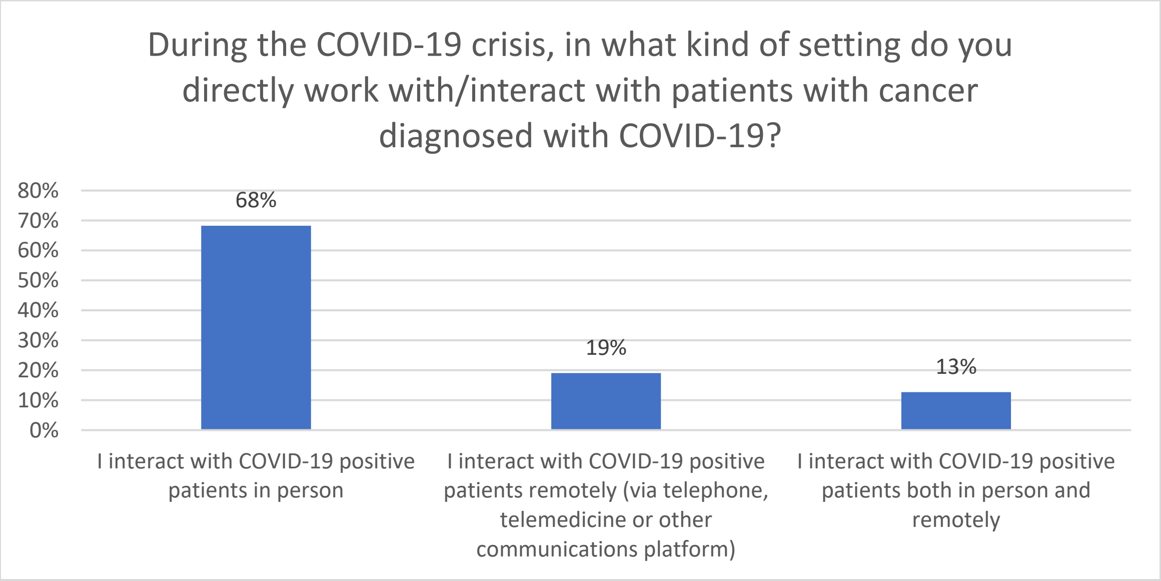

Table 3 Setting for interaction with patients with cancer diagnosed with COVID-19.
