# Supplementary material for: Staff Experiences at a New York City Medical Center During the Spring Peak of the Covid-19 Pandemic: A Qualitative Study
Source: Res Sq. 2021 Mar 24:rs.3.rs-268807. Preprint. [Version 1] doi: 10.21203/rs.3.rs-268807/v1 (PMC8010741; doi:10.21203/rs.3.rs-268807/v1)
Supplement: Supplement [file 60622d1dfa46384037217178.pdf]

Supplementary Information
Appendix A1 & A2

Article Title:
Staff Experiences at a New York City Medical Center During the Spring Peak of the Covid-19 Pandemic: A Qualitative Study

Journal Name:
Supportive Care in Cancer

Author Names:
Liz Blackler, MBE, LCSW-R, Amy E. Scharf, MBE, James N. Masciale, MA, Kathleen A. Lynch, MS MPH, Jamie C. Riches, DO, Konstantina Matsoukas, MLIS, Michelle Colletti, MS-PA-C,Lisa Wall, PhD, RN, CNS, HEC-C, Sanjay Chawla, MD, Nessa Coyle, NP, PhD, Yesne Alici, MD, Rebecca Guest, MD, MPH, Louis P. Voigt, MD, MBE

Corresponding Author:
Liz Blackler, MBE, LCSW-R
Memorial Sloan Kettering Cancer Center
Email: blacklel@mskcc.org

Appendix A1

Reflections

Given the nature of cancer care, the work we do during "normal" times at MSK can be difficult, distressing, and even traumatic. These experiences may be exacerbated or intensified by the COVID-19 pandemic. We want to hear about your reactions to these difficult moments, both expected and unexpected, and things you've found helpful for relieving stress at this time. In order to explore ways in which our faculty and staff have been impacted, and ways in which we can help, we're asking you to share your COVID-19 experiences.

The image below is cover from *The New Yorker* magazine from April 6, 2020, a time at which New York City's COVID-19 infection rate was rising. Your impressions and reactions to this image may help you reflect upon your experiences during this time and guide your answers to questions 1 and 2.

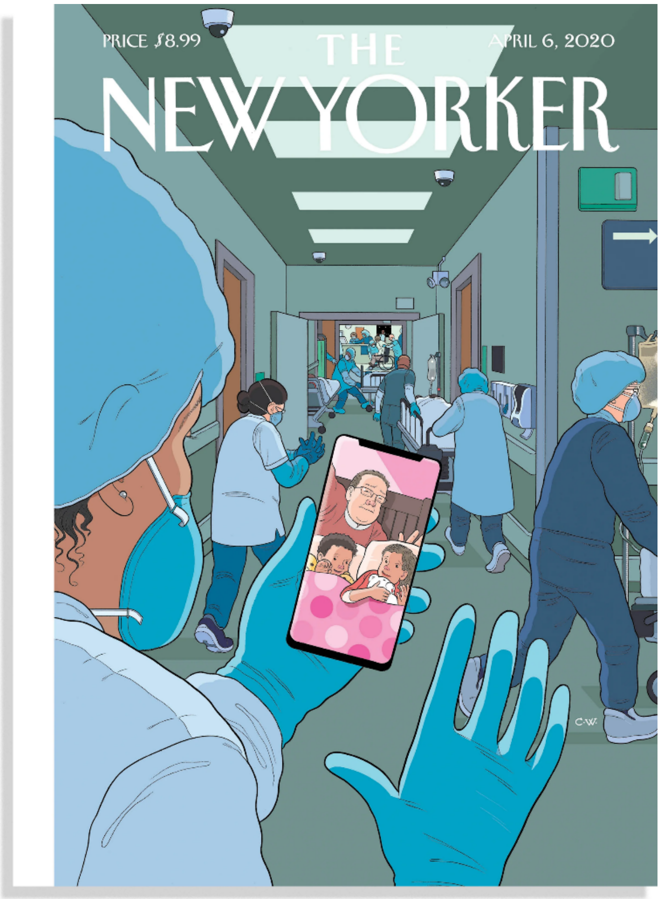

For these next 7 questions, please write **as much as you'd like** on:

Question #1:

Why do you do this work?

Question #2:

Please talk about the weight of the COVID-19 pandemic on you, personally.

Question #3:

For YOU, what has been the **most difficult** part of your work during the pandemic?

Question #4:

For YOU, what has been the **most gratifying** part of your work during the pandemic?

Question #5:

What have you found to be **helpful** in getting you through this challenging time?

Question #6:

What could MSK have done or do now to make this stressful period easier or more manageable for you?

Question #7:

Is there anything else you'd like to tell us?

Supplementary Information
Appendix A2

<u>Question 1 Sample Responses</u>

"I do this work because you become a part of a persons life while they are facing the impossible! Nobody wants to ever be told they are diagnosed with cancer. No person is ever diagnosed alone this affects their loved ones as well. I honestly love helping and being there for those during there most vulnerable times!"

"I want to make a difference in the world and have a strong desire to help people."

"Because I enjoy making a difference, even the smallest difference...whether it's holding a patient's hand when they're scared, or making sure they get their antibiotics, or making sure they understand what medications they're going home with and what they are used for. Also, because I enjoy helping people."

"To help others in their time of greatest need. Being a nurse is a passion, being an MSK nurse is a calling."

<u>Question 7 Sample Responses</u>

"I AM and will continue to remain to be a proud MSK employee whom is grateful to be associated to this institution. This is an amazing place to work for and I know those whom share the same pride and dedication I do...will continue to fight through these hard times in order to meet our departmental objectives and ultimately continue to provide great service to our patients!"

"MSK is blessed to be well resourced and with dedicated staff"

"Nutritionists need to be onsite. We cannot do any physical assessment of the patient from home. Working from home has been very stressful, feel no longer part of the medical team. Thank you!"

"I am grateful for the IT support we have gotten as employees; the IT team is amazing in their comprehensiveness and responsiveness to our needs and concerns (this was evident in helping us set up telemed and virtual desktop so

seamlessly). I understand there is software that can track if employees are truly online- I think that this would be helpful for those managers who are questioning if remote work is working or not."
